# Formaldehyde-Free Resins for the Wood-Based Panel Industry: Alternatives to Formaldehyde and Novel Hardeners

**DOI:** 10.3390/molecules27154862

**Published:** 2022-07-29

**Authors:** Justyna Chrobak, Jolanta Iłowska, Anna Chrobok

**Affiliations:** 1 Łukasiewicz Research Network—Institute of Heavy Organic Synthesis “Blachownia”, Energetyków 9, 47-225 Kędzierzyn-Koźle, Poland; jolanta.ilowska@icso.lukasiewicz.gov.pl; 2 Joint Doctoral School, Silesian University of Technology, Akademicka 2a, 44-100 Gliwice, Poland; 3 Department of Chemical Organic Technology and Petrochemistry, Faculty of Chemistry, Silesian University of Technology, Krzywoustego 4, 44-100 Gliwice, Poland; anna.chrobok@polsl.pl

**Keywords:** wood adhesives, formaldehyde-free resins, hardeners, ionic liquids

## Abstract

Due to its carcinogenic properties, the presence of formaldehyde in resins and other industrial products has been a subject of great concern in recent years. The presented review focuses on modern alternatives for the production of wood-based panels; i.e., substitutes for formaldehyde in the production of amino and phenolic resins, as well as novel hardeners for formaldehyde-free wood adhesives. Solutions in which formaldehyde in completely replaced are presented in this review. Recent advances indicate that it is possible to develop new formaldehyde-free systems of resins with compatible hardeners. The formaldehyde substitutes that have primarily been tested are glyoxal, glutaraldehyde, furfural, 5-hydroxymethylfurfural, and dimethoxyethanal. The use of such substitutes eliminates the problem of free formaldehyde emission originating from the resin used in the production of wood-based panels. However, these alternatives are mostly characterized by worse reactivity, and, as a result, the use of formaldehyde-free resins may affect the mechanical and strength properties of wood-based panels. Nonetheless, there are still many substantial challenges for the complete replacement of formaldehyde and further research is needed, especially in the field of transferring the technology to industrial practice.

## 1. Introduction

Nowadays, urea, melamine, and melamine–urea formaldehyde resins are still most commonly used in the production of particle boards. Formaldehyde emission from wood-based panels and furniture is, therefore, unavoidable, and an important issue, especially when these products are used indoors. The need to eliminate formaldehyde results from its presence, in free form, in the products, as well as its high toxicity. Manufacturers of wood panels have various options to meet the requirements set before them, including the use of modified urea–formaldehyde, melamine–formaldehyde, and phenol–formaldehyde resins with reduced formaldehyde emissions, or formaldehyde-free adhesives, such as para-methylene diisocyanate (pMDI).

This review focuses on presenting alternative solutions: resins in which formaldehyde is completely replaced by other compounds, and novel hardeners for formaldehyde-free resins.

## 2. Resins Used in the Wood-Based Panel Industry

Wood-based panels are widely used in furniture manufacturing and interior decorating. In general terms, the method of producing wood-based panels consists of coating wood chips with a suitable adhesive (a binder) and then arranging a form of the defined thickness and pressing it at a high temperature [1]. Under the influence of pressure and temperature, the glue binds the chips together. The hot product—the raw board—is then cooled and cut to selected size and thickness. Wood-based products include particleboard, plywood, oriented strand board (OSB), medium density fiberboard (MDF) and high-density fiberboard (HDF). Thermosetting resins are used as binders. Amino resins (urea–formaldehyde and melamine–formaldehyde), phenol–formaldehyde, isocyanate-based, and epoxy resins are the most common adhesives in the wood industry [2,3]. Urea– (UF), melamine– (MF) or melamine–urea– (MUF) formaldehyde resins are economically advantageous, and their synthesis and processing pose no technological problems, although the emission of formaldehyde is an inherent feature [4].

Urea–formaldehyde (UF) resins are typically used in the manufacture of wood-based products for interior applications, mainly particleboard and medium-density fiberboard. Wood boards manufactured with UF resins are, in general, characterized by limited moisture resistance, and emit detectable amounts of formaldehyde. These resins are also fast curing and exhibit good performance. However, the release of formaldehyde from products bonded with UF is a growing health concern [5].

Melamine–formaldehyde (MF) resins are used primarily for decorative laminates, paper treating and paper coating. Despite their high cost, these resins may be used in conventional wood-based composites, often in combination with UF, as their presence contributes to the reduction of the formaldehyde emission from wood-based panels [6].

Phenol–formaldehyde (PF) resins tend to be more chemically stable and less susceptible to hydrolysis than UF resins. They are typically used in the manufacture of construction plywood and oriented strand board for exterior applications. Phenolic resins are fast curing, but provide reduced mechanical properties and are expensive [7].

Isocyanate wood adhesive is a polymeric methylene di-isocyanate (pMDI). pMDI resins are typically more expensive than other resins, but cure easily and tolerate higher moisture content in the wood source. pMDI resins are sometimes used in core layers of composites, while PF resins are used in surface layers. These adhesives are prepared from methylenediphenyl diisocyanate (MDI), a chemical compound which, although it eliminates the problem of formaldehyde emission, is toxic to humans, causes respiratory and skin diseases, and is still being tested for mutagenicity and genotoxicity [8].

### 2.1. The Synthesis of Resins

**Figure 1:**
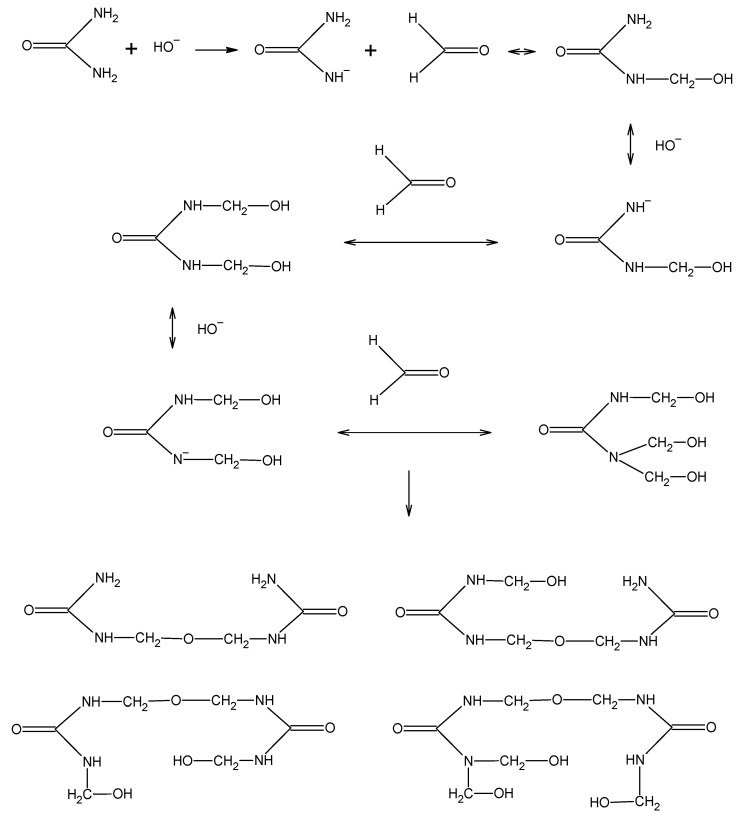
Alkaline condensation of urea and formaldehyde.

**Figure 2:**
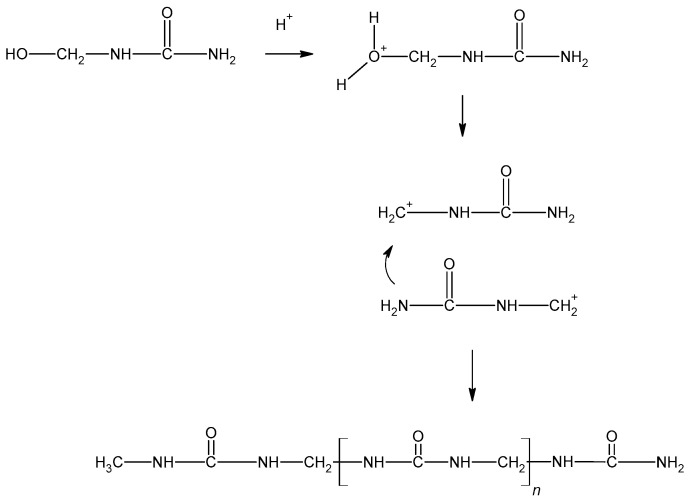
Copolymerization of monomethylolureas.

Urea–formaldehyde resins are primarily synthesized from urea and formaldehyde, with formaldehyde acting as the cross linker [9]. UF resins are prepared with a pH above 7 at the start of the reaction. The alkaline condensation leads to the formation of mono-, di- and trimethylolureas (Figure 1) [10]. Under alkaline conditions, the condensation is much slower and easier to control. The mixture is further reacted under acidic conditions, at about pH 5, and with heating, which leads to the desired degree of condensation. The acid condensation of the methylolureas induces the formation of firstly soluble and then insoluble cross-linked resins (Figure 2). Parameters that can alter the outcome include: pH, reaction time, reaction temperature, and formaldehyde/urea molar ratios at different stages of the reaction.

**Figure 3:**
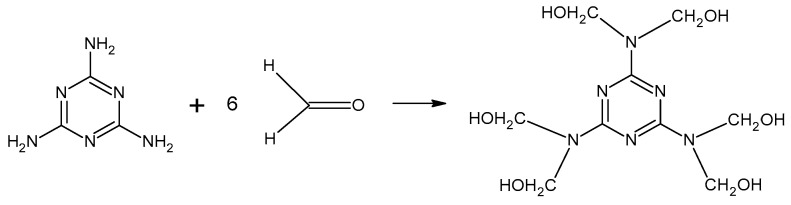
Reaction between melamine and formaldehyde.

The condensation of melamine with formaldehyde is the initial reaction in the formation of melamine–formaldehyde resin (Figure 3). The synthesis of resin is carried out at a pH between 6 and 9, which results in various methylol compounds with between two and six methylol groups attached [11]. Then, methylene and ether bridges form, which causes the molecular weight of the resin to increase rapidly.

**Figure 4:**
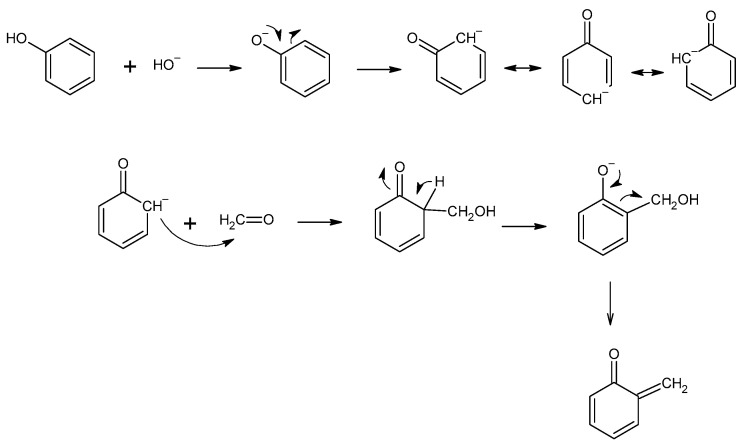
The formation of the quinone methide.

**Figure 5:**
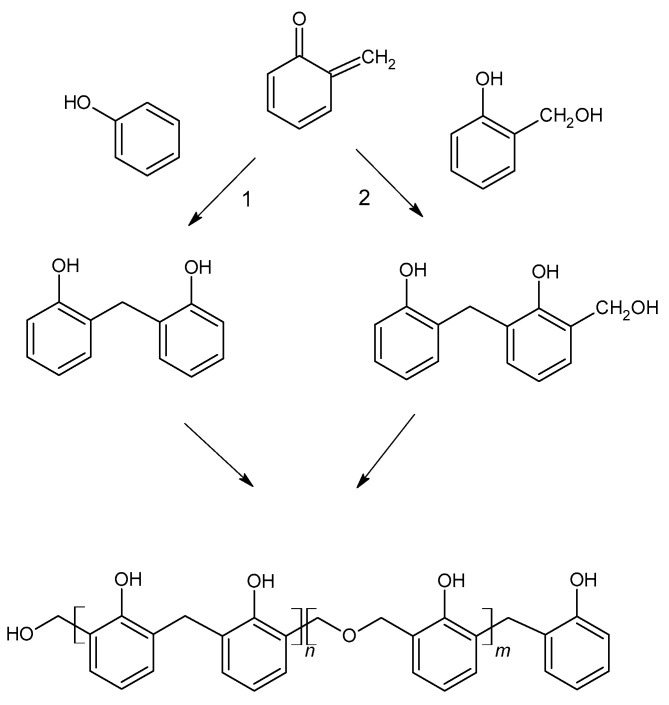
The formation of phenol formaldehyde resin.

Resol resins are synthesized in the reaction of phenol and an excess of formaldehyde (1.6:1 to 2.5:1) [10]. The phenoxide anion is the reactive molecule formed by deprotonation of phenol. Then, in the reaction with formaldehyde, a quinone methide is formed (Figure 4) and condensed to form resol. The condensation can occur in two ways, although the second reaction is favored (Figure 5). During heating, molecules containing methylol groups combine to create larger molecules, and eventually gel and form the solid-state resin [10].

### 2.2. The Hardening/Curing Process

**Figure 6:**
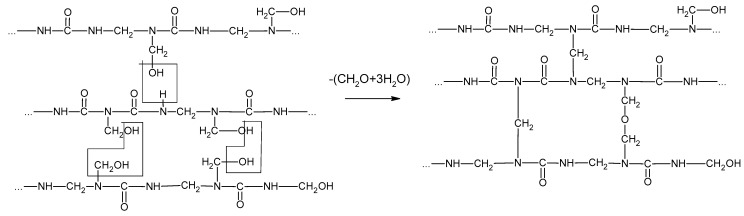
The curing process of urea–formaldehyde resin.

The curing of resins plays an important role in the gluing process, determining the quality and properties of the wood-based products. The curing process of UF resin condenses the liner structure of the resin into a ridged three-dimensional structure (Figure 6) [12]. The condensation reactions occur between adjacent polymers, with the adjacent nitrogen within the amide group forming methylene bridges, and between the methylol groups forming ether bridges. The curing of MF resins is similar to that of UF resins.

The core of the curing process is the enhancement of the acidity of the adhesive. Therefore, an acidic substance, or a substance capable of releasing acid when mixed with a resin, should be the curing agent (hardener). If the curing agent itself is a strong acidic substance, the pH of the glue solution decreases after the addition, and the rate of decline is too fast, which easily causes the pre-cure phenomenon of the glue.

Ammonium chloride, which can chemically react with formaldehyde to form an acidic substance to promote curing of the resin, is most commonly applied as a hardener of UF resin. It is an affordable and relatively inexpensive reagent, but makes the adhesive layer more fragile [13]. It is generally considered that the reaction of ammonium chloride with the free formaldehyde in UF resin yields acids during curing, which results in the acceleration of condensation reaction. The chemical reaction is presented as follows:

(1) $$4\;{\mathrm{NH}}_{4}\mathrm{Cl}+6\;H_{2}C=O\to 4\;H_{2}C=\mathrm{NH}+6\;H_{2}O+4\;\mathrm{HCl}$$

## 3. Toxicity of Formaldehyde

Formaldehyde is an organic chemical compound, the first in the homologous series of aldehydes. A 35–40% solution in water is the most common commercially available solution of formaldehyde, and is known as formalin. Formaldehyde is widely used as a key raw material in the industry of synthetic resins, adhesives, dyes, paints, and varnishes intended for various industries. It is also a preservative and disinfectant; therefore, it has been widely used in the production of cosmetics and hygiene products. In pharmacy, medicine, and biology, it is used as a preservative and fixative for medical and biological preparations [14].

The health risks of formaldehyde are a major concern, due to the widespread use of formaldehyde. People can be exposed to formaldehyde by inhaling gases emitted by formaldehyde-containing materials, or by absorbing it through the skin in liquid products. When formaldehyde enters the human body, usually through the respiratory system, it binds to iron-containing enzymes, in the form of formic acid, and causes cell hypoxia. It also leads to the multiplication of free radicals that contribute to the development of cancers. Specifically, formaldehyde exposure has been associated with myeloid leukemia, lung cancer, and nasopharyngeal cancer [15,16,17,18]. Research shows that the gas can cause allergies (inflammation of the skin) and aggravate respiratory problems, and can be very dangerous for people with asthma. Contact with the gas may cause irritation of the eyes, nose, and throat, inflammation, lacrimation, burning conjunctiva, sore throat, headaches, nausea, weakness, and insomnia. On the other hand, humans do not accumulate formaldehyde in the body—there is a gradual oxidation in the tissues, and the half-life of formaldehyde in human plasma is estimated to be 1–1.5 min.

According to the definition in the EU Regulation “Classification, Labelling and Packaging of substances and mixtures” (CLP) [19], since 2016 formaldehyde has been considered a substance with a category 1B carcinogenicity (i.e., a substance potentially carcinogenic to humans), and the classification is based on animal tests. Category 2 mutagenicity describes substances of concern in humans due to the potential to induce heritable mutations in the germ cells in humans; similarly, this classification is based on positive evidence obtained from experiments in mammals. The European Chemicals Agency (ECHA) has conducted, and is still conducting, a series of investigations/consultations on formaldehyde and formaldehyde-releasing substances and their use in the EU, so that stricter standards and restrictions can be introduced. This is also the view of the International Agency for Research on Cancer (IARC), which is the part of the World Health Organization (WHO), who in 2006 concluded that there was sufficient evidence that formaldehyde was “carcinogenic to humans” based on higher risks of nasopharyngeal cancer and leukemia [20].

The discussed classifications are reflected in the documents intended for the wood-based panel industry, which processes huge amount of various formaldehyde resins. The European standard EN 13986 has been in force since 2004 for wood products used in construction. The boards are classified into emission classes (E1–E3) depending on how much formaldehyde is released from them. To classify a board into an E1 class, it must not release more than 0.1 ppm under carefully established test conditions. The Environmental Protection Agency (EPA), from the USA, in its 2010 report included the information that formaldehyde is a “carcinogen to humans”, and 6 years later they issued a document defining the maximum emissions of formaldehyde from various types of wood-based panels, generally in the range 0.05–0.13 ppm.

Such classification of formaldehyde legitimately contributes to its negative perception, and is the reason for introducing regulations that limit the permissible emission levels of this compound. It is also the reason for working on alternative solutions. At the beginning of the 1970s, extensive research was conducted to reduce formaldehyde emissions from wood-based panels. The introduction of several methods, to industrial practice, of reducing the content of free formaldehyde from about 100 mg per 100 g of board to less than 8 mg per 100 g of board was the result of these studies, and was accomplished by changing the method of synthesis or by the use of various modifiers [21,22], which are substances that chemically bind formaldehyde, e.g., amines (urea, ammonia, melamine, and dicyandiamide), polyamines, sodium sulphite, or tannins [23]. Due to the ongoing work aiming to reclassify formaldehyde, more and more stringent standards regarding the emission of formaldehyde, and the strengthening of environmental awareness, it should be expected that its content and emissions from wood-based products will be further reduced in the future.

## 4. Substitutes for Formaldehyde in Formaldehyde-Free Resins in the Wood Industry

**Figure 7:**
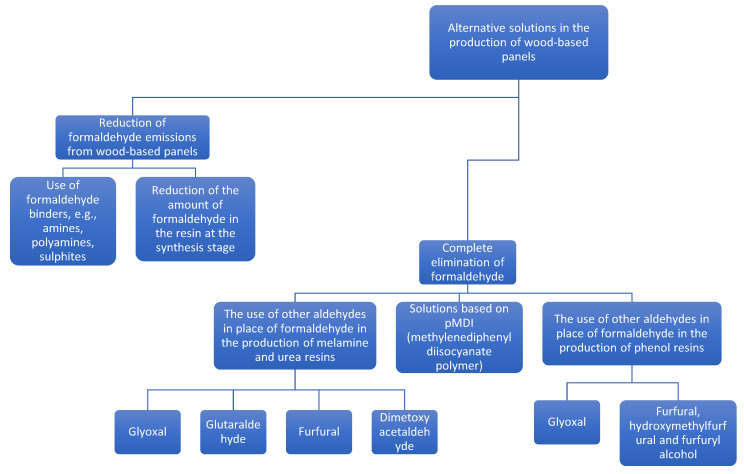
Summary of alternative solutions in the wood-based panel industry.

The need to reduce or eliminate formaldehyde from the production of various types of products results from its high toxicity and its presence, in free form, in the products. Unbound formaldehyde penetrates directly into the environment, while bound formaldehyde is released only as a result of resin degradation, which is influenced by high humidity and temperature. With increasingly restricting health and safety regulations, the complete elimination of formaldehyde and its substitution seems to be one of the most important methods of reducing the presence of free formaldehyde in the finished wood products, although in the end the technological performance of novel adhesive systems will be a decisive factor in introducing solutions to industrial practice [24]. The review [24] provides a detailed technological assessment of various adhesive systems focusing on the parameters crucial for wood-based panel industry. The summary of alternative solutions in the wood-based panel industry is presented in the Figure 7.

### 4.1. Amino (Urea and Melamine) Formaldehyde-Free Resins

The amino resins are the most important type of adhesives in the wood-based panel industry, mainly used for the production of particleboards and medium density fiberboard. The literature, in recent years, indicates the possibility of using alternative aldehydes for the synthesis of various types of resins, intended for various industries, including the wood-based panel industry (Table 1).

#### 4.1.1. Glyoxal

Due to its low toxicity, glyoxal, the simplest aliphatic dicarbonyl compound, is potentially an excellent substitute for formaldehyde. The LD50 of this compound is 3300 mg/kg (oral, rat) [30]. Glyoxal has already been used as a formaldehyde substitute in several wood-adhesive applications, and has been described in a few publications.

In 2014, Deng et al. used the nonvolatile and nontoxic glyoxal to prepare glyoxal−monomethylol–urea resins. These resins were characterized by FTIR and ^13^C NMR spectroscopies, and the basic properties and bonding strength of bonded plywood panels were tested, proving that they can be directly used as furniture material in dry conditions [25].

In 2014, Deng et al. synthesized urea−glyoxal resin under weak acid conditions and tested the bonding strength of the plywood. The resin was characterized by matrix-assisted laser desorption ionization—time of flight—mass spectrometry (MALDI-TOF-MS), and the mechanism of its creation was described. The results showed that the bonded plywood could be directly used as interior decoration and furniture material [26].

In 2017, Deng et al. synthesized melamine−glyoxal resins and characterized their structure with the use of ^13^C NMR and MALDI-TOF-MS. The energy of activation of cross-linking of these resins was measured, and it was found to be higher than that of melamine−formaldehyde resins, which could be a limitation in the practical use as binders in the wood industry [27].

In 2018, Xi et al. reported that melamine–glyoxal–glutaraldehyde resins are capable of bonding interior and exterior grade plywood industrially, under significant press conditions, when an ionic liquid is used as the hardener [28]. The melamine−glyoxal resins were prepared at molar ratio 1:6, and small proportions of glutaraldehyde were added to improve the water resistance of the wood panels. The structure of the resin was described with the use of analytical techniques (FTIR, MALDI).

Moreover, amino resins are one of the most important retanning agents used in the leather industry. In 2015, melamine−glyoxal resin was used as a replacement for the conventional formaldehyde-based melamine resin, with better tanning performance [31]. Urea−glyoxal or melamine−glyoxal resins can also be used to encapsulate volatile compounds (mainly odors) [32].

#### 4.1.2. Glutaraldehyde

In 2010, Mamiński et al. described the synthesis of a formaldehyde-free adhesive based on urea and glutaraldehyde. It was found that blending with nano-Al_2_O_3_ was necessary for satisfactory performance as an adhesive [33].

The use of glutaraldehyde in the synthesis of amine resins is more noticeable in the leather industry. Formaldehyde is used in the production of leather, which, in the end, causes the finished product to contain free formaldehyde. At present, tanners face a technical challenge to produce leather of high quality while meeting ecological and safety standards. Ecolabelling concepts have created awareness to produce formaldehyde-free leathers, and, for this purpose, several investigations have been carried out. In 2015, formaldehyde was replaced with glutaraldehyde, an industrially available aldehyde which is less toxic than formaldehyde [29]. Melamine–formaldehyde can successfully be used as a retanning agent, providing the required physicochemical properties, e.g., tensile and tear strengths, of retanned leathers.

#### 4.1.3. Furfural and 5-Hydroxymethylfurfural

5-Hydroxymethylfurfural is a promising bio-derived chemical with a broad scope of possible application, e.g., in the production of solvents, fuels, polymers, and adhesives [34].

In 2018, 5-hydroxymethyl furfural was used as a modifier to improve the properties of melamine−glyoxal resins. The prepared resin was tested as a plywood adhesive, and it was proven that the curing-activation energy of the resin was found to be lower than that of melamine−glyoxal resin [35].

In 2018, Zhou et al. described the use of furfural as a substitute for formaldehyde in the synthesis of melamine resin. The formaldehyde-free melamine resin showed excellent performances in leather processing [36]. The thickness and shrinkage temperature of leather were increased due to the rigid furan ring.

#### 4.1.4. Dimethoxyethanal

According to the literature, dimethoxyethanal (DME, dimethoxyacetaldehyde) is another compound which can be used in the synthesis of formaldehyde-free amino resins with the use of melamine or urea. DME is a non-volatile and non-toxic aldehyde, is a glyoxal derivative, and can be used in the production of wood-based panels. DME can be obtained from the controlled reaction of methanol with glyoxal under acidic conditions. Compared to glyoxal, in which two adjacent aldehyde groups provide a very high reactivity, DME exhibits the functionality of formaldehyde, containing one formyl group. Consequently, DME can replace formaldehyde in the production of melamine and urea resins. Similar to formaldehyde, DME can react with melamine and urea in similar pH ranges, but its level of reactivity is much lower.

In 2008, Despres et al. presented the synthesis of melamine−urea−DME resin with the use of glyoxylic acid [37]. The use of glyoxylic acid during the reaction allowed the formation of various oligomers, both by aldol condensation and by condensation of glyoxylic acid with two melamine molecules to form dimers. The structure of resins was determined with the use of MALDI-TOF and ^13^C NMR analysis. It was stated that these resins had not meet the requirements, so the addition of isocyanate (pMDI) was necessary to satisfy the relevant mechanical strength standards of panels.

In 2010, Properzi et al. presented the synthesis of dimethoxyethanal-derived resins and research on their performance as an adhesive. It was found that all formulations met the requirements of the current standards for class P2 particleboards for general uses. The authors pointed out the main advantages of DME (colorless, low toxicity, easy handling, and high stability at room temperature), but also concluded that the reactivity of the adhesive needs to be enhanced to fulfill the requirements of the wood industry [38].

In 2010, Despres et al. described urea−dimethoxyethanal resins and their usability in the wood industry. These resins were underperforming technically, due to the lower reactivity of DME in relation to formaldehyde, so the addition of 14% isocyanate (pMDI) was necessary to meet the requirements of the industry [39].

### 4.2. Phenolic, Lignin, and Tannin-Based Resins

Phenolic resins are mainly obtained from phenol, along with highly toxic and volatile formaldehyde. Various resins, especially those made from natural resources (tannins or lignins) [40] and substitutes of formaldehyde, have been used in the production of wood-based panels. Aldehydes derived from lignin were also reported as a potential replacement for formaldehyde in the synthesis of phenolic resins.

#### 4.2.1. Glyoxal

Tannins are natural compounds which can be used as a substitute for phenol due to their phenolic nature. In 2005, Ballerini et al. synthesized tannin−glyoxal resins, which were used to produce wood-based panels that do not contain formaldehyde. It was found that, in this case, glyoxal can be used as hardener, and the mechanical properties of panels can be improved by adding polymeric isocyanate (pMDI) [41].

Many phenolic resins were prepared by replacing phenol with lignin, which is an environment friendly material. Lignins are cheap phenolic materials, and have been the subject of many research papers since 1980s [42,43,44].

In 2007, Mansouri et al. presented the synthesis of lignin-based wood adhesives prepared without formaldehyde, which was replaced by glyoxal [45,46]. The adhesives provided good mechanical properties of the wood-based boards and showed sufficient reactivity.

In 2007, Lei et al. also prepared lignin-based resins with the use of glyoxal [47]. Internal bond strength of the panels was tested, and proved to be good enough to pass relevant international standard specifications for interior-grade panels. Satisfying properties of wood-based panels were also obtained in 2007 by Amaral-Labat et al. when glyoxalated soy flour was added to the glyoxalated lignin or tannin [48].

In 2010, mass spectrometry MALDI-TOF was used by Navarette et al. to evaluate lignin–glyoxal reactions [49].

In 2018, Ang et al. used glyoxalated alkali lignin–phenolic resin to improve the stability of Green jelutong (Dyera costulata) wood [50].

In 2019, Younesi-Kordkheili et al. synthesized phenol−lignin−glyoxal resin, evaluated its use in the production of particleboards, and assessed its physical and mechanical properties [51]. Ionic liquids were also used for the modification of lignin which in result accelerated the curing time and decreased the curing temperature of the resin [52].

In 2019, Aziz et al. used lignin which was prepared from coconut husks. The obtained kraft lignin and soda lignin were characterized by various techniques, and then used to synthesize resins with the use of glyoxal. These resins were intended for use as a wood adhesive [53]. A novel lignin-based wood adhesive was also prepared with the use of kenaf lignin and glyoxal, and characterized in 2019 [54].

In 2022, both petroleum-based phenol and formaldehyde were replaced by lignin and glyoxal by Siahkamari et al. [55]. The performance of the lignin–glyoxal adhesive was comparable to both lignin–formaldehyde and phenol–formaldehyde adhesives. The formaldehyde-free adhesive had a relatively high dry adhesion strength, but failed the wet adhesion test.

#### 4.2.2. Furfural, Hydroxymethylfurfural, and Furfuryl Alcohol

Moreover, lignin and furfural were proposed to replace phenol and formaldehyde, respectively. In the 1980s, Pizzi et al. described phenol–furfural resins [56], which were then characterized by various analytical methods [57]. In 2013, the synthesis of tannin–furfuryl alcohol resins was described [58].

Further research was conducted by Dongre et al. in 2015. They used lignin and furfural to replace phenol and formaldehyde in the preparation of resins in various conditions in order to find the optimum conditions [59].

In 2015, Zhang et al. described the synthesis of 5-hydroxymethylfurfural phenol resin. An environment-friendly curing agent (organosolv or kraft lignin) was used, and the curing mechanism and kinetics were studied [60]. In 2016, Zhang et al. continued the research on phenol−hydroxymethylfurfural resin. The resin was cured with the use of hexamethylenetetramine, and it was stated that it can be used to make green composites with zero formaldehyde emission upon heating [61]. In order to prepare a resin from sustainable resources, phenol could be replaced with hydrolysis lignin [62]. In another study, bisphenol A diglycidyl ether was also used to crosslink the phenol–hydroxymethylfurfural resin [63].

In 2019, Cheng et al. described the synthesis of phenol-substituted bio-oil–phenol–formaldehyde adhesives, using furfural as the crosslinking agent [64]. In addition, in 2019, Sui et al. described the synthesis of formaldehyde-free biobased phenolic resole resins: phenol–furfural–glucose, phenol–furfural, and phenol–glucose. The use of glucose and its transformation into 5-hydroxymethyl furfural, with the use of an acid catalyst, was presented, and a possible mechanism for the formation of the resin was also suggested [65].

#### 4.2.3. Other Aldehydes

In 2016, Santiago-Medina et al. described an adhesive based on purified pine bark tannin and aldehydes derived from lignin, namely vanillin and a dialdehyde derivative of vanillin. The structure of the oligomers was determined by matrix-assisted laser ionization desorption time-of-flight (MALDI-TOF) mass spectrometry. It was stated that these resins could be used for the production of wood-based particleboards [66].

In 2016, Foyer et al. used aromatic aldehydes, such as 4-hydroxybenzaldehyde, vanillin, and syringaldehyde, which were obtained by the depolymerization of lignins. These aldehydes were used as precursors for the synthesis of formaldehyde-free resol-type phenolic resins. They were not reactive enough; therefore, a method of functionalization was developed [67].

Additionally, in 2016, Foyer et al. presented a method of functionalization to turn the lignin-based aldehyde precursors 4-hydroxybenzaldehyde and vanillin into difunctional and reactive aromatic aldehyde precursors, which were successfully used for the synthesis of resol-type phenolic resins [68].

### 4.3. Isocyanate-Based Resins

Isocyanate-based wood adhesives are a separate group of adhesives which are inherently formaldehyde-free and they are widely described in literature [69]. The use of 4,4-diphenylmethane diisocyanate (pMDI) in the production of wood-based panels is already well established [70] but there are still possibilities in the field of hybrid adhesives combining formaldehyde-free resins with the addition of pMDI.

Several researchers studied the addition of pMDI to urea–formaldehyde resins [71,72]. In 2018, Younesi-Kordkheili and Pizzi described the effect of pMDI on the physical and mechanical properties of particleboards made with urea–glyoxal resin. It was stated that the addition of pMDI significantly accelerated the gel time and improved the mechanical properties of particleboard panels [73]. pMDI was also added to formaldehyde-free resins based on lignin (glyoxalated kraft lignin [48] and glyoxalated calcium lignosulfonate [45,46]).

## 5. Novel Hardeners for Formaldehyde-Free Resins in the Wood Industry

Resins, in their uncured form, do not have a practical application. Only its cross-linked form is an interesting example of a material with excellent application properties. In order to provide the resin with functional properties, it is subjected to cross-linking process, obtaining an insoluble and infusible product.

### 5.1. Ionic Liquids

Ionic liquids (ILs) are chemical organic compounds composed solely of ions, the melting temperatures of which are lower than the boiling point of water. Due to their versatile properties, non-volatility, and high thermal stability, ionic liquids could be used in industrial processes in a wide range of applications, particularly as replacements for conventional compounds, mainly catalysts or solvents. The potential of ILs is nearly unlimited, as they can be designed according to the specific application [74].

#### 5.1.1. For Epoxy Resins

Before experiments were begun on the hardening of the amine resins, several ionic liquids were tested as latent crosslinkers for epoxy resins. For the first time, in 2003, 1-butyl-3-methylimidazolium tetrafluoroborate [BMIM][BF_4_] was used for epoxy-resin curing by Kowalczyk and Spychaj [75]. In 2009, Rahmathullah et al. tested 1-ethyl-3-methylimidazolium dicyanamide [EMIM]N(CN)_2_ in the process of epoxy resin crosslinking [76]. In 2011, it was confirmed that N,N′-dioctadecylimidazolium iodide [DODIM][I] is able to promote crosslinking of the epoxy pre-polymer without the presence of an external curing agent [77]. In 2012, 1-ethyl-3-methylimidazolium chloride [EMIM][Cl] and a eutectic mixture of imidazole and choline chloride were tested in the crosslinking of epoxy resin [78], as well as five imidazolium liquid salts [BMIM][BF_4_], 1-butyl-3-methylimidazolium dicyanamide [BMIM][N(CN)_2_], and the 1-decyl-3-methylimidazolium salts: chloride [DMIM][Cl], tetrafluoroborate [DMIM][BF_4_], and dicyanamide [DMIM][N(CN)_2_] [79]. In 2019, diglycidyl ether of bisphenol A-type epoxy resin was crosslinked with [BMIM][BF_4_] alone and combined with conventional hardeners. IL accelerated the curing process in the presence of aromatic amine, and improved the thermal stability of the system [80]. In 2020, 1-butyl-3-methylimidazolium chloride [BMIM][Cl] was used for the hardening of epoxy resin and the miscibility, morphology, thermo-mechanical properties, and surface hydrophilicity were studied before and after the accelerated weathering test [81].

#### 5.1.2. For Formaldehyde-Free Amine Resins

According to the literature, the hardening of melamine resins, e.g., glyoxal resins, can be performed with the use of various substances. Ammonium salts (e.g., NH_4_Cl) are one of the most important and effective types of catalysts used to harden resins. Chromium nitrate is another compound which can be used for cross-linking. In this case, a gel time of about 3 min was observed, while the required temperature was as high as 150 °C [28]. It has been shown that the problems encountered during the curing of resins are caused by the higher activation energy of the cross-linking of melamine–glyoxal resins, compared to melamine–formaldehyde resins. Suitable gel times could only be achieved at temperatures higher than 150 °C. In the case of the wood-based panel industry, these conditions are too extreme, as the maximum temperature reached in the core of the chipboard being prepared should not exceed 110–120 °C.

To solve the problem of high activation energy in the curing of resins, in the last few years an attempt was made to use acidic ionic liquids to reduce the curing temperature of the resins and improve their bonding ability.

The use of Brønsted acidic ionic liquid as a new catalyst for urea–glyoxal resins was investigated by Younesi-Kordkheili and Pizzi in 2016 [82]. N-methyl-2-pyrrolidone hydrogen sulfate [HNMP][HSO_4_] was used in various quantities [83]. Wood-based panels were then prepared and their properties were measured. The results indicated that ILs can be used efficiently as a catalyst for urea–glyoxal resins. The particleboard panels had better mechanical properties compared to products made with the use of NH_4_Cl, and, most importantly, the gel time was accelerated with increasing IL content.

In 2018, the addition of N-methyl-2-pyrrolidone hydrogen sulphate was also tested as an adhesive hardener for the melamine–glyoxal–glutaraldehyde resins. The chemical structure of the resins was described with the use of instrumental analytical techniques. The role of ionic liquids was also presented. It was stated that ILs catalyze the hardening of amine resins, resulting in a decrease in hardening temperature, as well as in the energy of activation of the hardening. They also catalyze the reaction of aldehydes to yield aldol condensation. ILs also contribute to the demethylation of lignin of the wood [29].

In 2018, Xi et al. described the synthesis of melamine–glyoxal resin, modified with 5-hydroxymethyl furfural, and the preparation and assessment of a bonded plywood. Ionic liquid [HNMP][HSO_4_] was used as a hardener, in the amount of 5% resins solids by weight. The prepared plywood was characterized by good water resistance and bonding properties [35].

### 5.2. Non-Formaldehyde Hardeners for Tannin Resins (Methylolated Nitroparaffins, Hexamine)

Tannin-based resins are often cross-linked by formaldehyde in a polycondensation reaction in order to function as a wood adhesive. Formaldehyde could be replaced by other chemicals which are less toxic and do not release toxic vapor. In 2001, Trosa and Pizzi described the use of methylolated nitroparaffins as a hardener for tannin-based resin [84]. Tannin autocondensation is another method which enables its use in the production of wood-based panels [85].

Tannin–hexamine (hexamethylenetetramine) adhesives are one of the most popular systems, and they are already commercially available. The main advantage is that these adhesives do not release formaldehyde in decomposition reactions. It was proven that the wood-based products obtained using tannin–hexamine adhesives satisfy the requirements of both interior and exterior grade-standard specification [86,87]. Nonetheless, further research was conducted. In 2006, Pichelin et al. described the use of hexamine for the hardening of mimosa tannin resins [88]. In 2010, Moubarik et al. synthesized cornstarch–tannin adhesives without the use of formaldehyde as a hardener, which was replaced by hexamine. Plywood which was bonded with this adhesive exhibited excellent mechanical properties [89].

## 6. Conclusions

Formaldehyde emission is one of the most important issues in the wood-based panel industry. As health concerns have increased over the years, the consumers and producers of wood-based panels have become more aware of the associated risks. Therefore, the need to reduce or eliminate formaldehyde from the production of various types of products results mainly from its high toxicity, its current classification, and the possibility of the complete prohibition of its use in the future.

The elimination of formaldehyde from resin composition is one of the best solutions to the problem of its presence in the free/unbound form in the finished products. Literature data from recent years indicate the possibility of using alternative aldehydes for the synthesis of various types of resins. Glyoxal, glutaraldehyde, dimethoxyethanal, and furfural are the most promising substitutes for formaldehyde. Moreover, the performance of these novel resins, on a laboratory scale, seems to meet the requirements of the wood-based panel industry. However, many issues remain to be solved in the future, due to the inferior reactivity of the formaldehyde substitutes and possibly their higher cost.

On the other hand, hardeners play a central role in the efficient utilization of wood resources and in the production of wood-based panels. They should be compatible with the resin and not deteriorate the properties of the wood-based boards. The development of novel solutions in the wood-based panel industry is, therefore, connected with research in the fields of both resins and hardeners.

## Figures and Tables

**Table 1:** Substitutes of formaldehyde and properties of resins and plywood.

Substitute of Formaldehyde	Properties of the Resin	Properties of Plywood	Literature
Glyoxal	Appearance light yellow liquid Viscosity 41.0 mPa·s Nonvolatile solid content 54.7%	Dry shear strength 0.90 MPa	[25]
Glyoxal	Appearance light yellow liquid Viscosity 25.0 mPa·s Nonvolatile solid content 48.2%	Dry shear strength 0.98 MPa	[26]
Glyoxal	Appearance light yellow liquid Viscosity 65–500 mPa·s Nonvolatile solid content 48.8–51.6%	Dry shear strength 0.70–0.93 MPa	[27]
Glyoxal + glutaraldehyde	Viscosity 390–2890 mPa·s Nonvolatile solid content 48.5–49.0%	Dry shear strength 0.59–0.76 Mpa 24 h Cold water shear strength 0.63–1.48 MPa	[28]
Glutaraldehyde	Viscosity 1000 mPa·s Nonvolatile solid content 56.0–60.0%	Shear strength 2.83–5.91 MPa	[29]

## Data Availability

Not applicable.
